# Accurate and rapid discrimination of cigarette and household decoration material ash residues by negative chemical ionization TOFMS via acid-enhanced evaporation

**DOI:** 10.1038/s41598-020-62814-1

**Published:** 2020-04-02

**Authors:** Shujun Liu, Yuanyuan Xie, Ximing Song

**Affiliations:** 1 Liaoning Key Laboratory for Green Synthesis and Preparative Chemistry of Advanced Materials, College of Chemistry, Liaoning University, Shenyang, 110036 China; 2 Shenyang Fire Research Institute of MEM, Shenyang, 110034 China; 3 Key Laboratory of Separation Science for Analytical Chemistry, Dalian Institute of Chemical Physics, Chinese Academy of Sciences, Dalian, 116023 China

**Keywords:** Natural hazards, Chemistry

## Abstract

The detection and identification of cigarette ash in fire debris can be meaningful in fire investigations caused by burning cigarettes. In this work, a novel analytical method based on negative chemical ionization time-of-flight mass spectrometry (NCI/TOFMS) combined with a phosphoric-acid-enhanced evaporation strategy has been developed for the discrimination of cigarette ash samples (CAs) and common household decoration material ash samples (CHDMAs). A series of characteristic ions representing the acidified products HNCO and formic acid in the CAs were achieved, whose signal responses were enhanced with the help of mechanical agitation operation. To account for both the signal responses of the characteristic ions and acid corrosion of the ion source, the dynamic-purge gas was chosen to be 200 mL/min. The whole time for analysis was only 5 min, which is suitable for high-throughput measurements of large quantities of fire debris. As a result, a preliminary discrimination was achieved between the CAs and CHDMAs by virtue of the chemometric tool of principal components analysis (PCA) based on intensity differences of the characteristic ions. The results are encouraging and highlight the potential of NCI/TOFMS without complicated sample preparation steps for the accurate and high-throughput identification of cigarette ash on substrates in fire debris.

## Introduction

It is well known that arson leads to property damage, loss of life, and feelings of insecurity in the community. In many arson cases, accelerants such as ignitable liquids (ILs) are used to initiate or accelerate the fire. The most commonly used ILs are petroleum-based products like gasoline and diesel, as they are obtained easily and cheaply^1^. Therefore, the detection and identification of ignitable liquid residues (ILRs) in fire debris at arson scenes is a critical step in any fire investigation, which could provide useful information for investigators in cases where arson is suspected^2^. However, evidence evaluation is complicated by interference from pyrolysis products of the substrate materials present in a fire, bacterial degradation, weathering, and firefighting efforts if samples are not collected in time^1,3–5^.

To solve the above-mentioned problems, especially the matrix effects in the fire debris, many kinds of analytical techniques in combination with sample preparation methods have been developed for the analysis of ILRs in fire debris^3,6^. Gas chromatography-mass spectrometry (GC-MS) is the most widely used analytical technique for the determination of accelerants in fire debris, which is provided by the American Society for Testing and Materials International (ASTM International) standard as a guideline for the identification and classification of ILRs from fire debris^7^. At present, GC-MS has been widely applied for the study of acidified ILRs in fire debris^8^, analysis of the trace residues of gasoline combustion on different substrate materials^9^, determining the effect of temperature on the weathering of gasoline^4^, and even examining potential interference of body products and substrates to the identification of ILRs on worn clothing^10^. However, differences in the chromatographic conditions and columns can lead to variations in retention times for ILRs from one laboratory to another, making inter-laboratory comparisons of GC-MS profiles challenging. Furthermore, GC-MS is time consuming, which hinders the rapid information collection for arson investigators. Therefore, many novel analytical methods have been developed for the fast and accurate investigation of ILRs in fire debris^3,11–16^. Laser-induced breakdown spectroscopy (LIBS) has been employed for real time *in-situ* analysis and depth profiling, which can provide valuable information about fire debris that is complementary to the classification of the original sample components and combustion residues^11^. However, the laser-based spectroscopic techniques require expensive and complicated laser devices, which limits the utility of LIBS in practical applications at fire scenes. Headspace-mass spectrometry electronic nose (E-Nose) was proposed for the analysis of ILRs, including the investigation of fire suppression agents and weathering process on ILs with fast determination capabilities, no solvent and absorbent requirement, and easy operation^12–14,17,18^. To solve the problem of GC-MS being time-consuming, a new method using direct analysis in real-time mass spectrometry (DART-MS) without extraction was developed for the fast identification of ILs from substrates^15^. Recently, an alternative approach based on the ion mobility spectrometry sum spectrum (IMSSS) from headspace analysis was developed to analyze ILRs in fire debris^16^. The results show that IMSSS is capable of fast, objective, and easy interpretation of fire debris data, real-time monitoring, and operation at the fire scene because the devices are portable. However, the fire debris samples have to be kept in the auto-sampler oven for agitation and heating for 20 min leading to time-consuming headspace processing step.

Sample preparation is a critical component in the analysis of samples. It is a step that is often scrutinized and challenged by the courts. Along with the analytical methods mentioned above, there are generally sample preparation procedures for extracting and concentrating volatile organic compounds (VOCs) from ILRs with complicated substrate materials in fire debris^3,6^. The passive headspace concentration with activated charcoal specified by the ASTM E1412 standard is currently the most commonly used method in the United States to isolate and concentrate VOCs from ILRs in fire debris because it is sensitive, easy to conduct, and non-destructive^19^. Solid-phase micro-extraction (SPME) using Tenax as a sorbent is another commonly used sample preparation technique^8,20^. Many new sample preparation techniques have been established, such as headspace single-drop microextraction (HS-SDME)^21^, negative pressure dynamic headspace concentration^22^, and headspace sorptive extraction (HSSE) with polydimethylsiloxane stir bars as the adsorbent phase^23^. New adsorbent materials were also synthesized and investigated for the concentration of volatile compounds in ILRs in fire debris^24,25^.

Data modification and chromatographic alignment are often utilized for accurate data interpretation for GC-MS^26–28^. In addition, chemometric tools are almost mandatory to identify and classify ILRs easily in less time, including principal component analysis (PCA)^29^, soft independent modeling of class analogy (SIMCA)^30^, partial least squares-discriminant analysis (PLS-DA)^28^, hierarchical cluster analysis (HCA)^20,31^, and a self-organizing feature map (SOFM) artificial neural network^32,33^.

In addition to ILRs, another ignition or accelerating material gaining growing attention is burning cigarettes, because cigarettes are daily supplies, easily obtained due to low prices, and have been frequently used by arsonists. Cigarette ends, which indicate the brand name and potentially carry smokers’ DNA, have long been an aspect of criminal investigations^34^. However, there are generally no cigarette ends but a small amount of cigarette ash samples (CAs) at the fire scene. It has been reported that there are a variety of constituents in cigarette smoke, such as organic compounds, inorganic acids, and trace metals^35–38^. However, most volatile compounds would evaporate from the cigarette ash during a fire because of high temperature and weathering. Many researches have been reported for assessing important role the cigarette ash may play in terms of trace metal distribution towards human health and environmental pollution^39–43^. The cigarette ash has been reported to retain 65 to 75% of the toxic metals Cd, Ni, Pb, and Cr through microwave digester and flame atomic absorption spectrophotometer (FAAS) detection^43^. More importantly, the trace-metal distribution of the cigarette ash has been employed for classification of tobacco brands in areas of forensics and criminology^44,45^. Jourdan *et al*. explored the trace metals in cigarette ash and yielded good specificity for classification of different cigarette brands by virtue of trace-metal concentrations^45^. Besides, non-volatile, polar organic compounds in cigarette ash samples were also investigated to differentiate three cigarette brands for forensic purposes based on GC-MS/MS^46^. What’s more, inorganic acid radicals were simultaneously detected beside the trace metals in the cigarette ash such as Cl and Br^47^. Therefore, the acid radicals inherent in the cigarette ash accompanying the trace metals may be used for distinguishing the cigarette ash from other material ash residues.

Recently, negative chemical ionization (NCI) based on an ion mobility spectrometer was applied for the rapid analysis of inorganic acid oxidizers in black powder, firecrackers, and match heads with O_2_^−^(H_2_O)n as the reactant ion^48^. As a nontraditional ionization mode, NCI has been employed for detecting various compounds, such as carboxylic acids, isocyanic acid, hydrogen cyanide, and other inorganic acids using negative reactant ions O_2_^−^, OH^−^, NO_2_^−^, O^−^, and I^−^ ^49–53^. The combination of an NCI ion source and time-of-flight mass spectrometry (TOFMS) may be a feasible solution for the analysis of acid radicals in ash samples.

In this paper, a method based on NCI/TOFMS in conjunction with a phosphoric-acid-enhanced evaporation process was firstly applied for fast acquisition of information on the acid radicals inherent in the ash samples without complicated extraction or adsorption steps. The product ions of the acidified products for the acid radicals and ionization mechanism are discussed. The flow rate of dynamic-purge sampling gas and solid-liquid mixture mode were investigated for the sensitive detection of CAs. The method was applied for the discrimination of CAs and common household decoration material ash samples (CHDMAs).

## Results and Discussion

### Ionization mechanism and characteristic spectral peaks for evaporated gas of acidified cigarette ash

**Figure 1:**
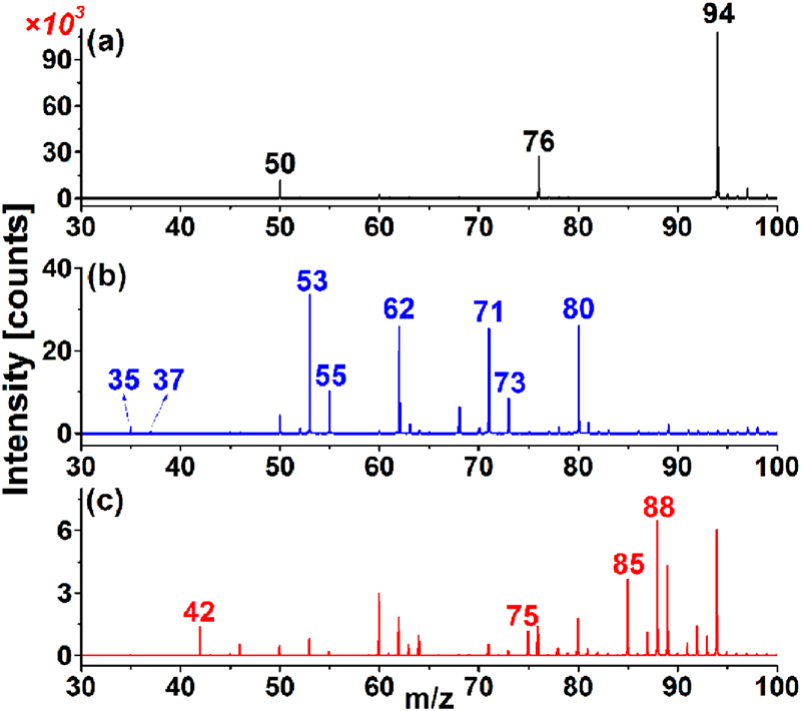
Mass spectra of evaporated gases of (**a**) 1 mL purified water, (**b**) 1 mL 2% H_3_PO_4_, and (**c**) 10 mg 1# cigarette ash with 1 mL 2% H_3_PO_4_, obtained by NCI/TOFMS (NCI/TOFMS: negative chemical ionization time-of-flight mass spectrometry).

Figure 1 shows mass spectra of the evaporated gases of purified water, 2% H_3_PO_4_, and acidified 1# cigarette ash with a purging air flow rate of 0.2 L/min. The characteristic ions of the purified water vapor gas were ions at *m/z* = 50, 76, and 94, which were attributed to H_2_O·O_2_^−^, CO_2_·O_2_^−^, and H_2_O·CO_2_·O_2_^−^, respectively. In Fig. 1(b), mass spectral peaks for the 2% H_3_PO_4_ vapor gas were not only at *m/z* = 50, 76, and 94 but also at *m/z* 53, 55, 62, 71, 73, and 80, corresponding to H_2_O·^35^Cl^−^, H_2_O·^37^Cl^−^, NO_3_^−^, 2H_2_O·^35^Cl^−^, and 2H_2_O·^37^Cl^−^ after calibration (See Table S1), which revealed the presence of HCl and HNO_3_ in H_3_PO_4_. However, characteristic ions of the evaporation gas for the acidified cigarette ash with H_3_PO_4_ included ions at *m/z* = 42, 75, 85, and 88 with considerable signal intensities, which were not observed in the mass spectra of purified water and H_3_PO_4_ vapor (Fig. 1(c)). Isocyanic acid (HNCO) has been reported in the smoke of burning nitrogen-containing materials, such as biomass, cooking, cigarette smoking, and even light duty diesel vehicle exhaust^54–56^. Therefore, the characteristic ions at *m/z* = 42, 75, 85, and 88 were identified as the ions NCO^−^, HNCO·O_2_^−^, HNCO·NCO^−^ and [HCOOHNCO]^−^, respectively, after the mass-to-charge calibration in Table 1, all of which were relevant to HNCO. Actually, there are few reports on the analysis of HNCO in combustion ash residues, let alone concentration differences in different combustion ash residues. Most of produced HNCO in the cigarette ash was suspected to be in the form of acid radicals after combustion because the HNCO molecules would volatilize into the smoke with a relatively high temperature. In this paper, HNCO in the form of acid radicals was thought to be converted into corresponding volatile HNCO molecules after acidification with H_3_PO_4_ and then ionized by NCI. In the NCI source based on ^63^Ni, many low-energy electrons were first produced. Reactant ions O_2_^−^ were formed through electron-capture reactions due to high electron affinity of the O_2_ molecule, which was combined with H_2_O and CO_2_ molecules in the sample gas, resulting in the production of cluster ions H_2_O·O_2_^−^ (*m/z* 50), CO_2_·O_2_^−^(*m/z* 76), and H_2_O·CO_2_·O_2_^−^ (*m/z* 94), as shown in Eq. 1–4 below. Subsequently, HNCO was ionized by the O_2_^−^ relevant cluster ions, and NCO^−^ was produced because of the higher gas-phase acidity of HNCO (${\Delta {\rm{rH}}}_{{\rm{m}},({\rm{HNCO}})}^{^\circ }$= 1427.5 ± 2.6 kJ/mol) than that of HO_2_ (${\Delta {\rm{rH}}}_{{\rm{m}},({{\rm{HO}}}_{2})}^{^\circ }$= 1476.9 ± 3.0 kJ/mol)^57^, as shown in Eq. 5. Above all, the association reactions between HNCO, HCOO^−^, NCO^−^ and the O_2_^−^ reactant ion were the predominant ionization processes, thereby producing HNCO·O_2_^−^ (*m/z* 75), HNCO·NCO^−^ (*m/z* 85), and [HCOOHNCO]^−^ (*m/z* 88) with high intensities, as depicted in Eq. 6–8.

1 $${O}_{2}+{e}^{-}\to {O}_{2}^{-},$$

2 $${O}_{2}^{-}+{H}_{2}O\to {H}_{2}O\cdot {O}_{2}^{-},$$

3 $${O}_{2}^{-}+C{O}_{2}\to C{O}_{2}\cdot {O}_{2}^{-},$$

4 $${O}_{2}^{-}+{H}_{2}O+C{O}_{2}\to {H}_{2}O\cdot C{O}_{2}\cdot {O}_{2}^{-},$$

5 $${O}_{2}^{-}+HNCO\to NC{O}^{-}+H{O}_{2},$$

6 $${O}_{2}^{-}+HNCO\to HNCO\cdot {O}_{2}^{-},$$

7 $$NC{O}^{-}+HNCO\to HNCO\cdot NC{O}^{-},$$

8 $$HCO{O}^{-}+HNCO\to {[HCOOHNCO]}^{-}.$$

**Table 1:** Characteristic ions of the evaporated gas of the acidified cigarette ash.

Measured mass [*m/z*]	Theoretical mass [*m/z*]	Mass error [ppm]	Characteristic ions
41.99628	41.99799	−40.7	NCO^−^
74.99572	74.99566	0.8	HNCO·O_2_^−^
85.00231	85.00382	−17.8	HNCO·NCO^−^
88.00573	88.00348	25.6	[HCOOHNCO]^−^

### Sensitivity enhancement with appropriate flow rate of dynamic-purge sampling gas and mechanical agitation

**Figure 2:**
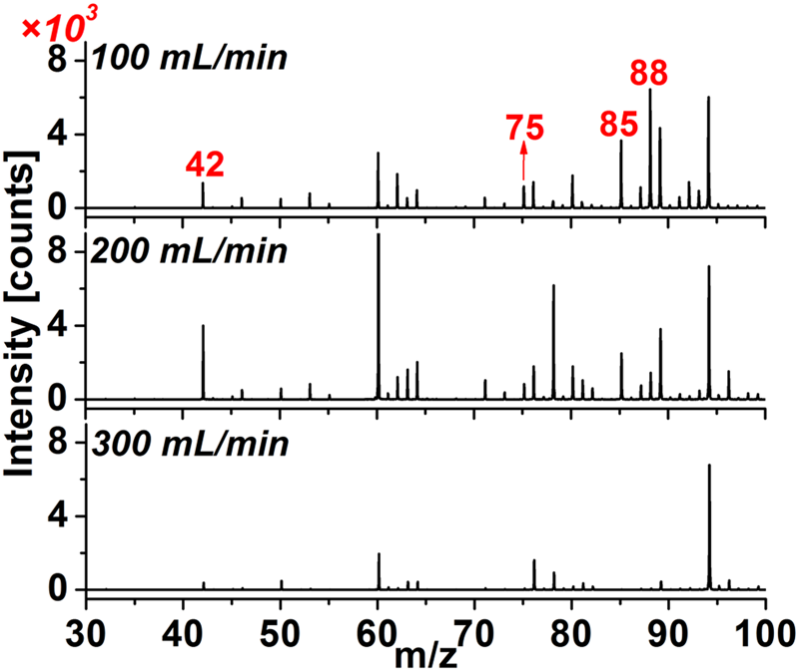
Mass spectra of the evaporated gas of 10 mg acidified 1# cigarette ash with different flow rates of dynamic-purge sampling gas.

In the present NCI/TOFMS method, the dynamic-purge sampling gas served to purge the acidified product gas into the NCI source for ionization. Increasing the flow rate of the dynamic-purge sampling gas would reduce interference to the ionization of HNCO and acid corrosion to the ion source by the volatile HCl and HNO_3_ in H_3_PO_4_ liquid. Meanwhile, the concentration of HNCO molecules in the NCI ion source would be diluted. Figure 2 shows the results for 10 mg 1# cigarette ash acidified with 1 mL 2% H_3_PO_4_ at different flow rates of the dynamic-purge sampling gas. The peak height of the parent ion NCO^−^ (*m/z* 42) increased from 1364 to 4004 counts, those of HNCO·O_2_^−^ (*m/z* 75) and HNCO·NCO^−^ (*m/z* 85) displayed no significant change while that of the ion [HCOOHNCO]^−^ (*m/z* 88) slightly decreased when the flow rate of the dynamic-purge sampling gas increased from 100 to 200 mL/min. The dilution of formic acid dominated. However, the peak heights of all ions decreased significantly when the flow rate of the dynamic-purge gas was 300 mL/min. the dilution of HNCO and formic acid both dominated. Considering reduction of the acid corrosion to the ^63^Ni-based NCI source with higher flow rate of the dynamic-purge sampling gas, the flow rate of 200 mL/min was selected for the following experiments.

**Figure 3:**
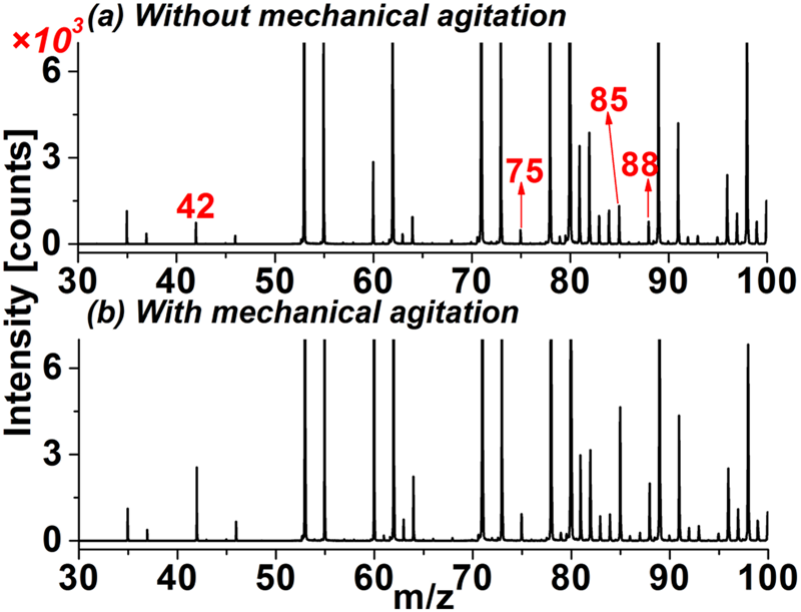
Mass spectra of the evaporated gas of the acidified 1# cigarette ash (**a**) without and (**b**) with mechanical agitation.

The characteristic ion responses of the evaporated gas for the acidified cigarette ash were not only relevant to the flow rate of the dynamic-purge sampling gas but also the mixture degree of the ash samples with 1 mL H_3_PO_4_. The mass spectra of the evaporated gas for the acidified 1# cigarette ash without and with mechanical agitation are illustrated in Fig. 3(a,b), respectively. Without mechanical agitation, the peak height of the characteristic ions NCO^−^ (*m/z* 42), HNCO·O_2_^−^ (*m/z* 75), HNCO·NCO^−^ (*m/z* 85), and [HCOOHNCO]^−^(*m/z* 88) were only 743, 485, 1321, and 777 counts, respectively. With mechanical agitation, the peak height of the above characteristic ions increased to 2553, 928, 4647, and 1995 counts, respectively, leading to enhancements of 1.9–3.5 times. Therefore, the mechanical agitation operation was utilized in the next experiments for better detection sensitivity.

### Discrimination of CAs and CHDMAs

**Figure 4:**
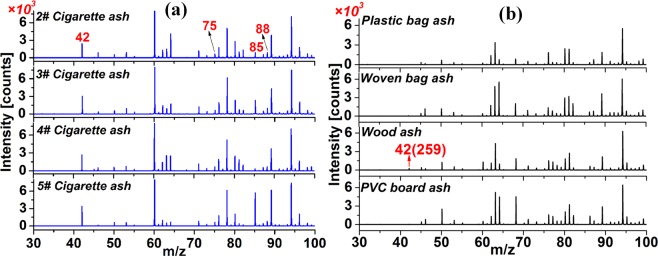
Mass spectra of the evaporated gases of (**a**) four acidified different brands of CAs and (**b**) four acidified different CHDMAs. (CAs: cigarette ash samples; CHDMAs: common household decoration material ash samples).

To investigate the discrimination performance of the developed technique of NCI/TOFMS for the CAs and CHDMAs, 31 different brands of CAs and 14 different types of CHDMAs were studied. The mass spectra of four different brands of CAs and four CHDMAs are displayed in Fig. 4(a,b), respectively. The characteristic ions NCO^−^ (*m/z* 42), HNCO·O_2_^−^ (*m/z* 75), HNCO·NCO^−^ (*m/z* 85) and [HCOOHNCO]^−^(*m/z* 88) were all detected with relatively high intensities for the four different CAs (Fig. 4(a)) but hardly detected for the four CHDMAs (Fig. 4(b)), which concluded that the ions above could be employed for the discrimination of CAs and CHDMAs. Results of other 26 different brands of CAs and ten different CHDMAs (in Fig. S1, S2, S3, and S4, respectively) confirmed the above conclusion and revealed the potential power of this method for the classification of CAs and CHDMAs. Although some of the characteristic ions were also observed in the mass spectra of the CHDMAs (wood ash in Fig. 4(b) and Fig. S4), the intensities were much lower than a tenth of those detected for the CAs. The results may be explained by the fact that the cigarette ash contained more nitrogen-containing compounds, such as nicotine and tobacco tar than other materials, which could produce more isohydrocyanate and formate after combustion with adequate oxygen^58^.

**Figure 5:**
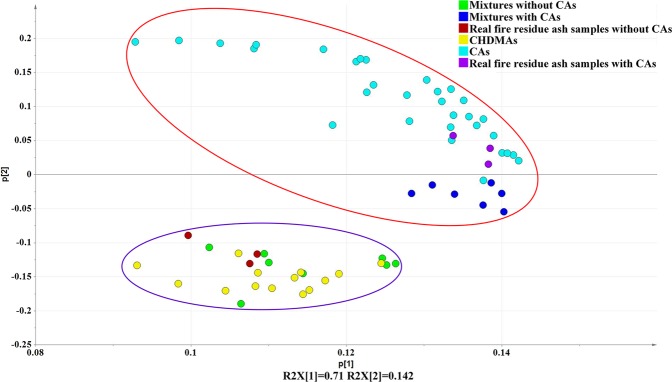
Scores plot with PC [1] and PC [2] of all samples studied.

To provide a direct and rapid interpretation of the data, a chemometric analysis based on PCA was carried out to visualize the variance caused by the acid ingredients in the acidification of evaporated gases of the ash samples. Considering that three-dimensional scores plot with PC [1], PC [2] and PC [3] failed to provide direct and rapid discrimination with the PC [3] score of only 2.88% (Fig. S5), scores plot with PC [1] and PC [2] was provided, as shown in Fig. 5. 85.2% of the variance of all the data was calculated because scores of PC [1] and PC [2] were 71% and 14.2%, respectively. The data points naturally clustered into two different groups: one related to the 31 CAs (sky blue dots, inside the red ellipse), and the other related to 14 CHDMAs (yellow dots, inside the purple ellipse). The PCA plots reinforce the conclusion above that the characteristic ions for the acidification products of the ash samples had the most significant effect on the discrimination of the CAs and CHDMAs.

### Identification of cigarette ash in common household decoration material ash and real fire residues in fire debris

In Fig. 5, the data points of seven ash mixtures by the CAs and CHDMAs (deep blue dots) are also contained in the cigarette ash cluster, while the other eight ash mixtures without any CAs (green dots) but only CHDMAs were contained in the CHDMA cluster. This showed that ash mixtures with even a few CAs were able to be differentiated with interference from the substrate materials. Furthermore, real fire residue ash samples from three different fire scenes were tested to further investigate the identification of the CAs in fire debris. Three real fire residue ash samples with CAs (purple dots) were observed in the CA cluster, while the other three real fire residue ash samples without any CAs (red dots) were contained in the CHDMA cluster, which proves that the inorganic compounds in the ash sample we focus on show no significant changes whether in the combustor temperature of 25 °C or in the high temperature of the fire. The data points representing the real fire residue ash samples with CAs even overlapped with those of some CAs, which illustrates promising identification tendency of the CAs in fire debris.

In summary, classification of the CAs and CHDMAs using PCA models based on the data obtained by NCI/TOFMS and phosphoric-acid-enhanced evaporation was satisfactory, with an overall classification rate of 85.2%. This was achieved even with the interference of different substrates, including real fire residues.

## Conclusions

In this study, NCI/TOFMS combined with phosphoric-acid-enhanced evaporation strategy was firstly used to differentiate CAs from CHDMAs with O_2_^−^ as the reactant ion. The analysis time was only 5 min without complicated extraction and chromatographic separation. The characteristic ions with significant intensity responses were produced for the CAs after acidification, and identified as the acidification products of HNCO and formic acid. A flow rate of 200 mL/min for the dynamic-purge sampling gas and mechanical agitation operation were chosen to enhance the signal responses of the characteristic ions. In combination with the chemometric tool of PCA, the NCI/TOFMS method was capable of discriminating the CAs and CHDMAs, and identifying the presence of the CAs in any of the studied scenarios. In conclusion, this novel method was preliminarily applied to discriminate the CAs and CHDMAs, which shows promising potential for the accurate identification of cigarette ash in fire scenes and auxiliary deduction of the fire cause.

## Methods

### Instrumentation

**Figure 6:**
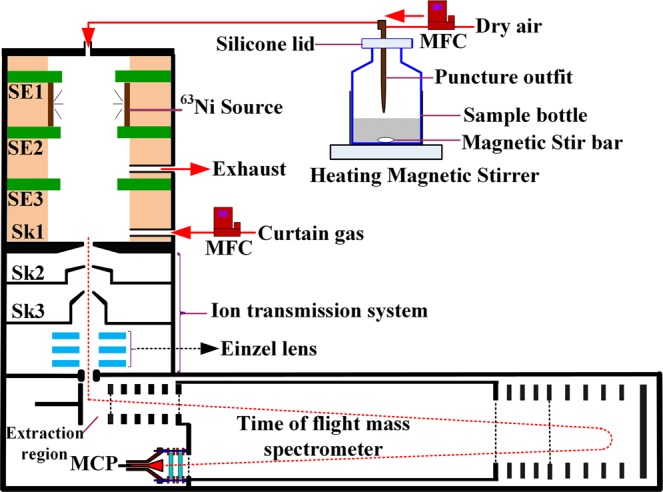
Schematic diagram of negative chemical ionization time-of-flight mass spectrometry (NCI/TOFMS) with phosphoric-acid-enhanced evaporation (SE: stainless steel electrode; Sk: skimmer; MFC: mass flow controller; NCI/TOFMS: negative chemical ionization time-of-flight mass spectrometry).

The homemade mass spectrometer shown in Fig. 6 consisted of a radioactive ^63^Ni-based negative chemical ionization (NCI) source, an ion transmission system, and an orthogonal acceleration TOF mass analyzer. The NCI source was constructed by a radioactive ^63^Ni source and an NCI region operated at atmospheric pressure. The NCI region included three 1-mm-thick stainless-steel electrodes (10 mm inner diameter (i.d.) center hole): SEI, SE2, and SE3. The radioactive ^63^Ni source was placed between electrodes SE1 and SE2. The three electrodes and a Skimmer 1 (Sk1) electrode were separated by four 10-mm-thick polytetrafluoroethylene (PTFE) insulation washers (12 mm i.d. center hole). Another 3-mm-thick PTFE insulation washer (12 mm i.d. center hole) was inserted between the electrode SE1 and the uppermost stainless-steel wall of the NCI ion source. The two PTFE insulation washers between the SE2 and Sk1 electrodes were set with a 3-mm i.d. side hole for gas flow. The side hole between the electrodes SE2 and SE3 was used for exhausting gas. The other side hole located closely above the Sk1 electrode was used to introduce a curtain gas to protect the radioactive ^63^Ni from acid corrosion and the orifice on the Sk1 from being blocked with ash particles. Another 3 mm i.d. hole designed in the middle of the uppermost stainless-steel wall was employed as the inlet for dynamically purging evaporated gas of the acidified ash samples. The flow rates of the dynamic-purge sampling gas and the curtain gas were controlled at 200 and 500 mL/min via two mass-flow controllers (Beijing Sevenstar Electronics Co., Ltd), respectively. The total length of the ^63^Ni-based NCI source from the uppermost stainless-steel wall to the Sk1 electrode was 36 mm. To maximize the ion transmission efficiency in the ion source, the negative direct current voltages from the electrodes SE1, SE2, and SE3 were −800, −600 and −400 V, respectively. A radio frequency quadrupole ion guide system would lead to significant mass discrimination for ions with small mass-to-charge ratios (*m/z*), such as NCO^−^ (*m/z* = 42), thereby decreasing the detection efficiency of HNCO. Thus, an electrostatic ion transmission system was employed in this study. The electrostatic ion transmission system was composed of three Skimmer electrodes and a set of electrostatic Einzel lenses. The distances between the orifices of Sk1, Skimmer2 (Sk2, 1 mm i.d. orifice), and Skimmer3 (Sk3, 1 mm i.d. orifice) were 2.4 and 3.4 mm, respectively. The ions in the ion source were transferred through the Skimmer orifices into the Einzel lens, which further collimated the ion beam and guided the ions through a 2 mm × 12 mm slit into a V-shape reflection TOF mass analyzer. A chevron MCP detector with a 50 Ω conical anode was used to collect the ions, and the TOF mass spectra were accumulated for 5 min using a 100-ps time-to-digital converter (TDC) (model 9353, Ametec Inc., Oak Ridge, U.S.A.) at a repetition rate of 40 kHz. A mass resolving power of 1900 (fwhm) at *m/z* = 94 was achieved, and all of the data were obtained by averaging the results from three measurements. The chambers between Sk2 and Sk3 and the Einzel lens chamber were differentially pumped by an 80 L/s (Hipace 80, Pfeiffer Ltd., Germany) and a 300 L/s (Hipace 300, Pfeiffer Ltd., Germany) turbomolecular pump, respectively. The TOF mass analyzer chamber was pumped by another 300 L/s turbomolecular pump (Hipace 300, Pfeiffer Ltd., Germany). The vacuum of the chamber between Sk1 and Sk2 was maintained by a 7.6 L/s rotary pump (E2M28, Edwards Ltd., U.K.), which was further used as the backing pump for the above turbomolecular pumps. This configuration enabled a high vacuum pressure (10^−4^ Pa) in the mass analyzer region while maintaining the pressure in the region between Sk1 and Sk2 electrodes at about 110 Pa. The instrumental parameters are summarized in Table S2.

### Dynamic-purge sampling system and phosphoric-acid-enhanced evaporation strategy

As shown in Fig. 6, the dynamic-purge sampling system included a mass flow controller, a heated magnetic stirrer, a 20 mL sample bottle with a silicone lid and a polyetheretherketone (PEEK) puncture outfit with one gas inlet and outlet. The mass flow controller was used for control of the flow rate of the purging gas. The sample bottle was heated to 40 °C for the evaporation promotion of the acidified products. Phosphoric acid (H_3_PO_4_) was used for extraction and acidification of the acid radicals in the ash samples because the NCI source was not susceptible to corrosion using H_3_PO_4_ with a low volatility^48^. The acid radicals could be extracted from the ash samples in the form of gaseous acid products, which enhanced the evaporation of the acid radicals with H_3_PO_4_. Most of all, there was no competitive ionization reaction between H_3_PO_4_ and the acidified products compared to the volatile hydrochloric acid (HCl) and nitric acid (HNO_3_), which eliminated overlaps between the characteristic ion peaks and background peaks. The acidified products were released from the ash sample and purged into the NCI source by dry air with a flow rate of 200 mL/min. Background TOF signals of the room air were recorded for 30 s to achieve background peaks at *m/z* 50 (H_2_O·O_2_^−^), 76 (CO_2_·O_2_^−^), and 94 (H_2_O·CO_2_·O_2_^−^) for calibration. Next, 10 mg ash sample was loaded into the sample bottle, 1 mL 2% H_3_PO_4_ was injected, and the heating magnetic stirrer began to operate. Immediately, the puncture outfit was pierced through the silicone lid for the gas flow, whose tail end was above the ash sample to prevent ash particles from being purged into the NCI source. The data acquisition was continuous for 5 min as soon as introduction of the acidified evaporation gas into the NCI source.

During the whole analysis process, the NCI source was maintained at 50 °C to reduce sample adsorption onto the source inner wall and prevent the PTFE insulation washers from deformation at higher temperatures. A new puncture outfit was used to eliminate the interference of sample residues on the next sample analysis.

### Sample preparation

Analytical grade 85% phosphoric acid (Kermel, Tianjin, China) and purified water (Wahaha, Hangzhou, China) were purchased and used to prepare 2% H_3_PO_4_ solutions. Thirty-one different brands of cigarettes were ignited by an igniter with static natural combustion and extinction without any control operation in a big combustor (size: 7 × 10 × 11 m^3^) with adequate oxygen and room temperature of about 25 °C. The corresponding produced CAs were numbered with 1# to 31# (Table S3), respectively. Nine CHDMAs (including cardboard, newspaper, carpet, wallpaper, white paper, plastic bag, woven bag, wood, and PVC board) were prepared in the same way as the CAs, while other five CHDMAs (including wall skin, red brick, powdered coal, cement, and ceramic tile) were ground to powder for analysis. Fifteen ash mixtures were prepared with some of the aforementioned CAs and CHDMAs by grinding each constituent with same mass together to investigate the classification power of this method. Seven of the ash mixtures each contained a different brand of cigarette ash, and the detailed information of the ash species of the fifteen ash mixtures is shown in Table S4. Three different real fire residue ash samples were obtained from three different real fire scenes caused by electrical damage. The three ash mixtures labelled as purple dots in Fig. 5 were respectively prepared by the above real fire residue ash samples and CAs with the same mass for each component to preliminarily identify CAs in fire debris.

### Chemometric analysis by principal component analysis (PCA)

PCA is generally used for identifying patterns in data by highlighting their similarities and differences. The original data set is described by means of new variables known as principal components (PCs), which are derived from linear combinations of the original variables with specific loadings for each principal component. A score plot of the first two PCs is most commonly used to display the cluster outcomes of a given data set, where samples with similar scores are positioned closely together. This plot provides an important means of visualizing and summarizing the original data set and often reveals patterns that were previously elusive^32^. In this paper, necessary data processes were carried out before the PCA: first, the intensity data from *m/z* = 30 to 100, which contained most of the mass spectral peaks, were extracted and divided by the intensity of a background peak at *m/z* = 76 (CO_2_·O_2_^−^) for the calibration of the slight intensity fluctuation. Second, the normalized intensity data for three background peaks at *m/z* = 50 (H_2_O·O_2_^−^), 76 (CO_2_·O_2_^−^, and 94 (H_2_O·CO_2_·O_2_^−^) with relatively high intensities were set to zero to eliminate their interference in the PCA results. Finally, the SIMCA 13.0 software was used for PCA analysis of the processed data for visual classification of different ash samples.

## Supplementary information

Supplementary information.
