# Supplementary material for: Accurate and rapid discrimination of cigarette and household decoration material ash residues by negative chemical ionization TOFMS via acid-enhanced evaporation
Source: Sci Rep. 2020 Apr 2;10:5810. doi: 10.1038/s41598-020-62814-1 (PMC7118106; doi:10.1038/s41598-020-62814-1)
Supplement: Supplementary file 1 — Supplementary information. [file 41598_2020_62814_MOESM1_ESM.docx]

**Supporting information**

**Accurate and rapid discrimination of cigarette and household decoration material ash residues by negative chemical ionization TOFMS via acid-enhanced evaporation**

Shujun Liu,^1,2^ Yuanyuan Xie,^3^ Ximing Song^1,^*

^1^Liaoning Key Laboratory for Green Synthesis and Preparative Chemistry of Advanced Materials, College of Chemistry, Liaoning University, Shenyang, 110036, China

^2^Shenyang Fire Research Institute of MEM, Shenyang, 110034, China

^3^Key Laboratory of Separation Science for Analytical Chemistry, Dalian Institute of Chemical Physics, Chinese Academy of Sciences, Dalian, 116023, China

Address reprint requests to Ximing Song, Liaoning Key Laboratory for Green Synthesis and Preparative Chemistry of Advanced Materials, College of Chemistry, Liaoning University, Shenyang, 110036, China.

*Tel*: +86-24-62202378; *Fax*: +86-24-62202380; *e-mail*: lsjsy7812@163.com

**Table S1.** Characteristic ions of the evaporation gas for 2% H_3_PO_4_

| Measured Mass [*m/z*] | Theoretical Mass [*m/z*] | Mass Error [ppm] | Characteristic ions |
| --- | --- | --- | --- |
| 52.98115 | 52.97942 | 32.6 | H_2_O·^35^Cl^−^ |
| 54.97753 | 54.97647 | 19.3 | H_2_O·^37^Cl^−^ |
| 61.98758 | 61.98783 | −4.0 | NO_3_^-^ |
| 70.98868 | 70.98999 | −18.4 | 2H_2_O·^35^Cl^−^ |
| 72.98576 | 72.98704 | −17.5 | 2H_2_O·^37^Cl^−^ |
| 79.9980 | 79.9984 | −5.0 | H_2_O·NO_3_^−^ |

**Table S2.** Instrumental parameters of the reflectron TOFMS (TOFMS: time-of-flight mass spectrometry)

| Ionization source | ^63^Ni |
| --- | --- |
| Skimmer 1 | −20 V |
| Skimmer 2 | −13 V |
| Skimmer 3 | −5 V |
| Lens 1  Lens 2  Lens 3 | +50 V  +464 V  +454 V |
| Extracting electrode V_pulse_ | ±400 V, 40 kHz |
| Accelerating electrode V_acc_ | +3545 V |
| Retarding electrode V_ref1_ | +362 V |
| Reflecting electrode V_ref2_ | −495 V |
| MCP V_mcp_ | +6000 V |
| Ionization region length | 35 mm |
| Extraction region length | 10 mm |
| Acceleration region length | 44.5 mm |
| Reflecting region length | 111 mm |
| Field-free-drift region length | 348.5 mm |
| MCP detector | *Φ*36 |

**Table S3.** Information of 31 different brands of cigarettes.

| Number | Cigarette brand | Manufacturer |
| --- | --- | --- |
| 1 | Zhongnanhai (Cool cool fashion) | Shanghai tobacco group co. LTD |
| 2 | Yun (Secret Garden) | Hongyun honghe tobacco group co. LTD |
| 3 | Nise | Hunan China tobacco industry co. LTD |
| 4 | Mild Seven | Shanghai made Japan tobacco industry co. LTD |
| 5 | Lan Zhou | Gansu tobacco industry co. LTD |
| 6 | Great Hall of the People (thin) | Hongta liaoning tobacco co. LTD |
| 7 | Chinese Caterpillar Fungus | Inner Mongolia kunming cigarette co. LTD |
| 8 | Chungwa | Shanghai tobacco group co. LTD |
| 9 | Double Happiness | Guangdong China tobacco industry co. LTD |
| 10 | Gold Leaf (large gold circle) | Henan China tobacco industry co. LTD |
| 11 | Great Hall of the People | Hongta liaoning tobacco co. LTD |
| 12 | Sue | Jiangsu China tobacco industry co. LTD |
| 13 | Peony | Shanghai tobacco group co. LTD |
| 14 | Huang Shan (first grade) | Anhui China tobacco industry co. LTD |
| 15 | Changbai Mountain | Jilin tobacco industry co. LTD |
| 16 | Mount Tai | Shandong China tobacco industry co. LTD |
| 17 | Gui | Guizhou China tobacco industry co. LTD |
| 18 | Lotus King | Hunan China tobacco industry co. LTD |
| 19 | Diamond (rose) | Hebei China tobacco industry co. LTD |
| 20 | Diamond | Hebei China tobacco industry co. LTD |
| 21 | Yellow Crane Tower | Hubei China tobacco industry co. LTD |
| 22 | Dragon (jin 'an) | Heilongjiang tobacco industry co. LTD |
| 23 | Pride | Sichuan China tobacco industry co. LTD |
| 24 | Yuxi | Red tower tobacco group co. LTD |
| 25 | Mount Tai (Buddha's light) | Shandong China tobacco industry co. LTD |
| 26 | Forever Daqianmen | Shanghai tobacco group co. LTD |
| 27 | Lotus | Hebei China tobacco industry co. LTD |
| 28 | Yun Cigarette (hulun buir) | Hongyun Honghe tobacco group co. LTD |
| 29 | Huang Guoshu (long march. Red star shining) | Guizhou China tobacco industry co. LTD |
| 30 | Panda | Shanghai tobacco group co. LTD |
| 31 | Jiangshan | Tianjin cigarette factory |

**Table S4.** Information of fifteen ash mixtures.

| Number | Mixed ash species |
| --- | --- |
| 1 | wallpaper, wall skin, carpet |
| 2 | wallpaper, wall skin, carpet, **Double happiness cigarette** |
| 3 | newspaper, red brick, cardboard, cement |
| 4 | newspaper, red brick, cardboard, cement, **Huang Shan (first grade) cigarette** |
| 5 | newspaper, red brick, ceramic tile, carpet, wallpaper, powdered coal, wall skin, white paper |
| 6 | newspaper, red brick, ceramic tile, carpet, wallpaper, powdered coal, wall skin, white paper, **Dragon (jin 'an) cigarette** |
| 7 | woven bag, plastic bag, wall skin |
| 8 | woven bag, plastic bag, wall skin, **Panda cigarette** |
| 9 | PVC board, wood, woven bag |
| 10 | PVC board, wood, woven bag, **Lotus cigarette** |
| 11 | woven bag, plastic bag, wall skin, wood, PVC board, newspaper, white paper |
| 12 | woven bag, plastic bag, wall skin, wood, PVC board, newspaper, white paper, **Mount Tai cigarette** |
| 13 | powdered coal, white paper, ceramic tile |
| 14 | white paper, wall skin, carpet, powdered coal, cardboard |
| 15 | carpet, ceramic tile, cardboard, newspaper, wood, wallpaper, **Yuxi cigarette** |


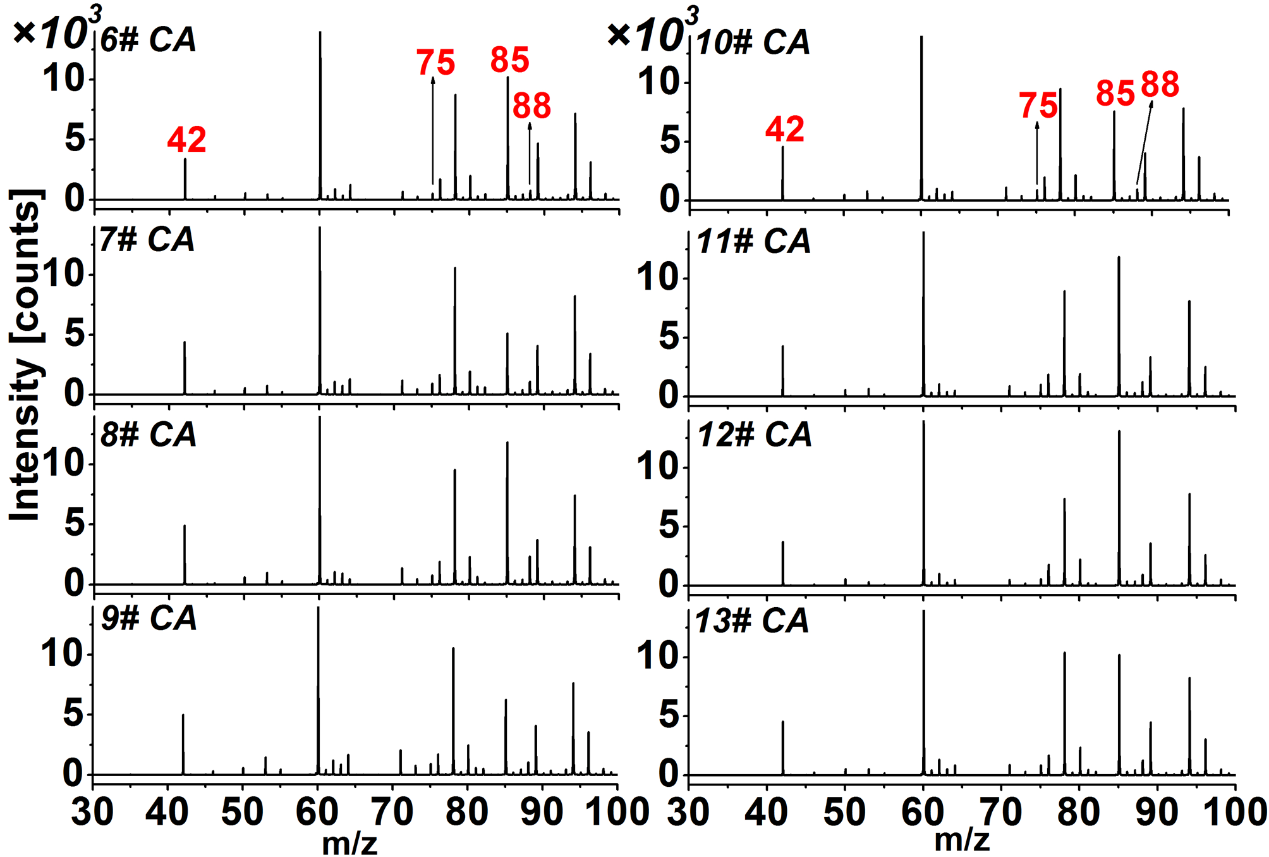


**Figure S1.** Mass spectra of the evaporated gases of eight acidified brands of CAs by NCI/TOFMS (6#, 7#, 8#, 9#, 10#, 11#, 12# and 13# CAs) (CAs: cigarette ash samples; NCI/TOFMS: negative chemical ionization time-of-flight mass spectrometry)


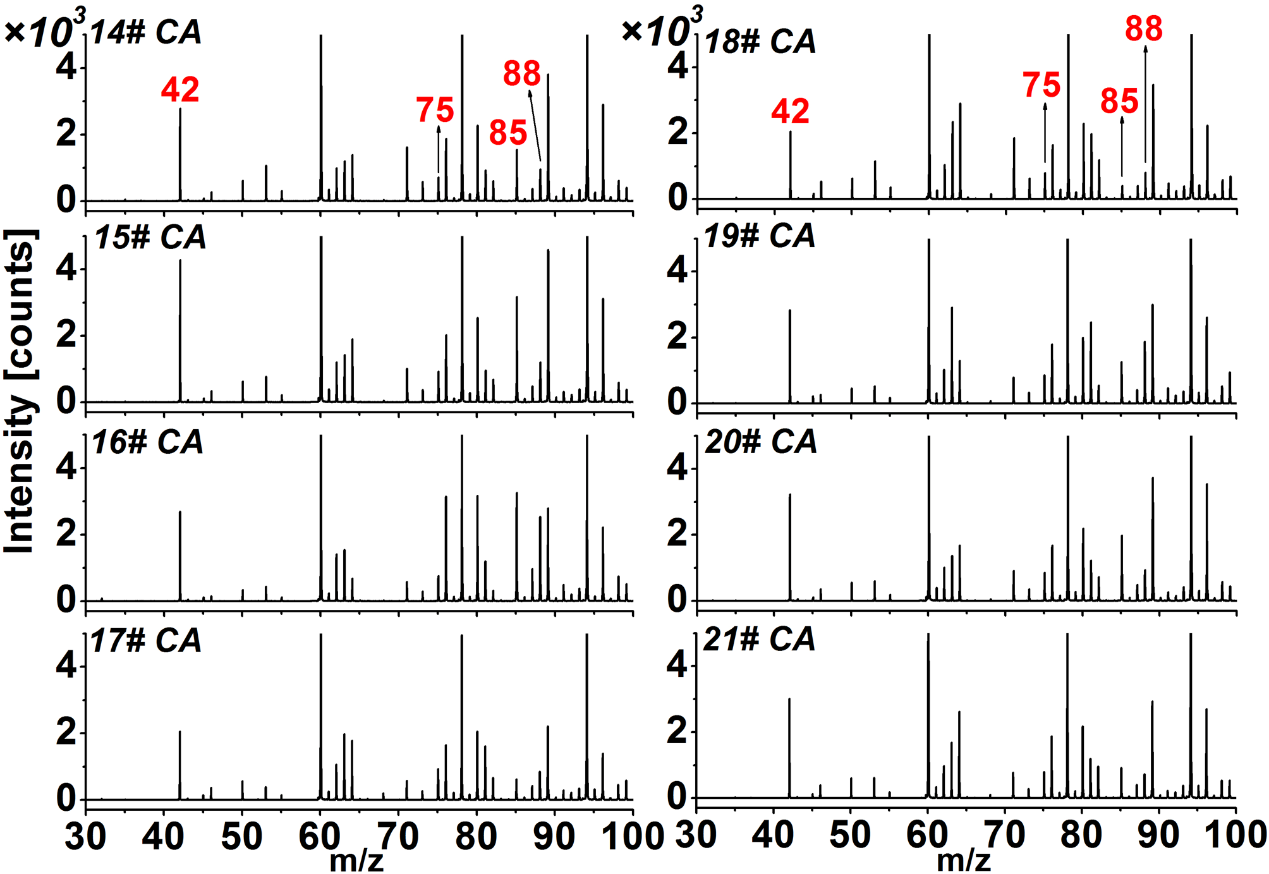


**Figure S2.** Mass spectra of the evaporated gases of eight acidified brands of CAs by NCI/TOFMS (14#, 15#, 16#, 17#, 18#, 19#, 20# and 21# CAs) (CAs: cigarette ash samples; NCI/TOFMS: negative chemical ionization time-of-flight mass spectrometry)


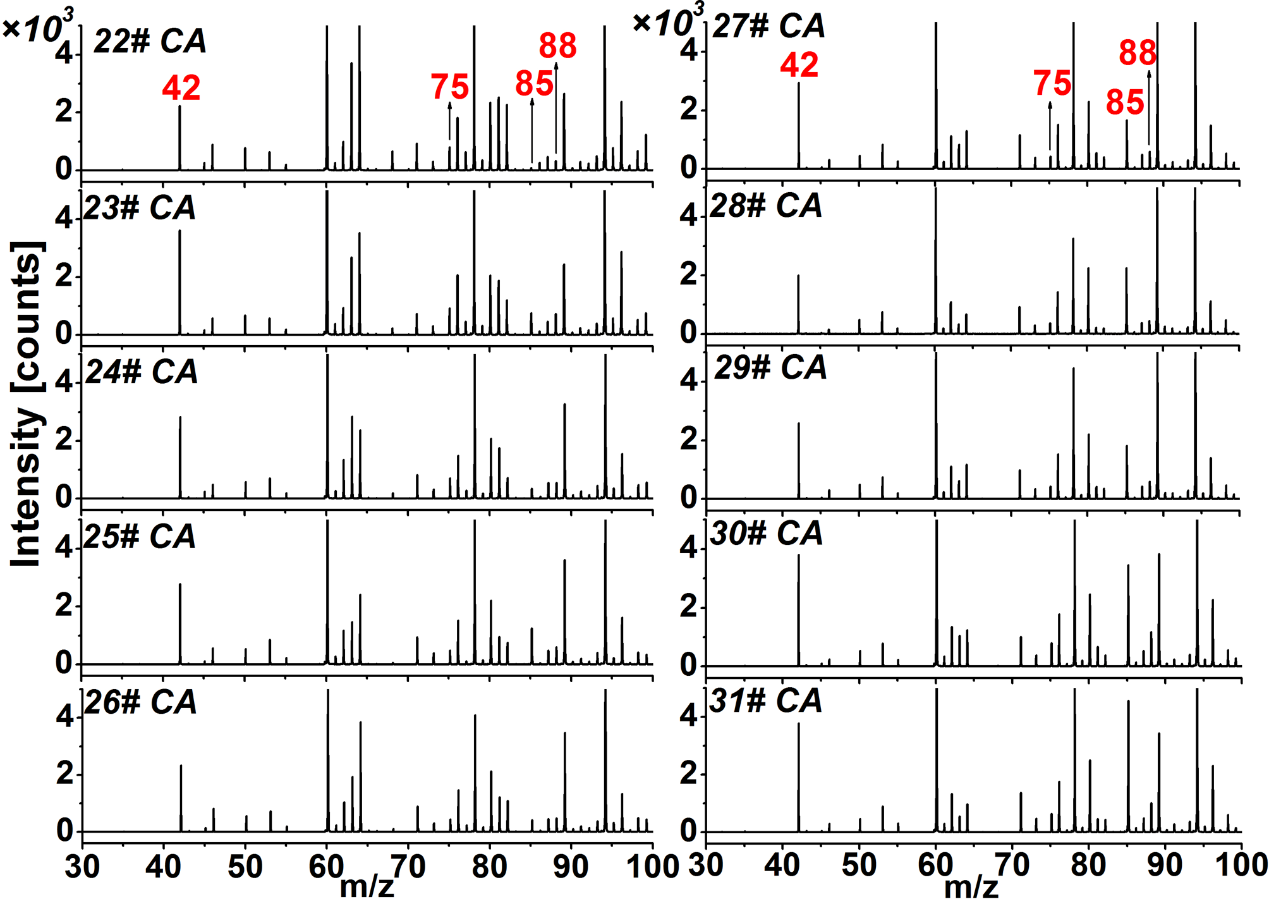


**Figure S3.** Mass spectra of the evaporated gases of ten acidified brands of CAs by NCI/TOFMS (22#, 23#, 24#, 25#, 26#, 27#, 28#, 29#, 30# and 31# CAs) (CAs: cigarette ash samples; NCI/TOFMS: negative chemical ionization time-of-flight mass spectrometry)


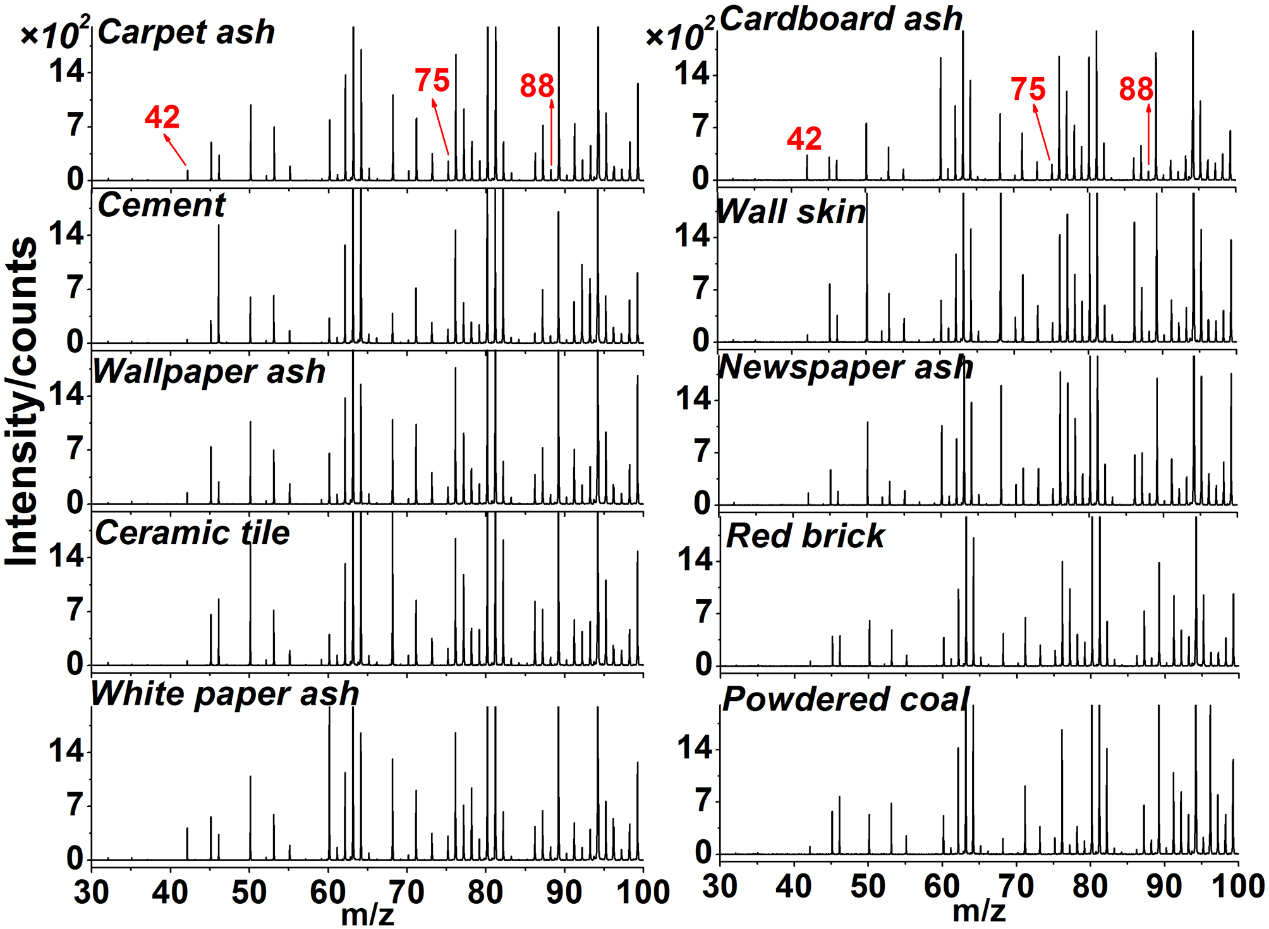


**Figure S4.** Mass spectra of the evaporated gases of ten acidified CHDMAs by NCI/TOFMS (CHDMAs: common household decoration material ash samples; NCI/TOFMS: negative chemical ionization time-of-flight mass spectrometry)


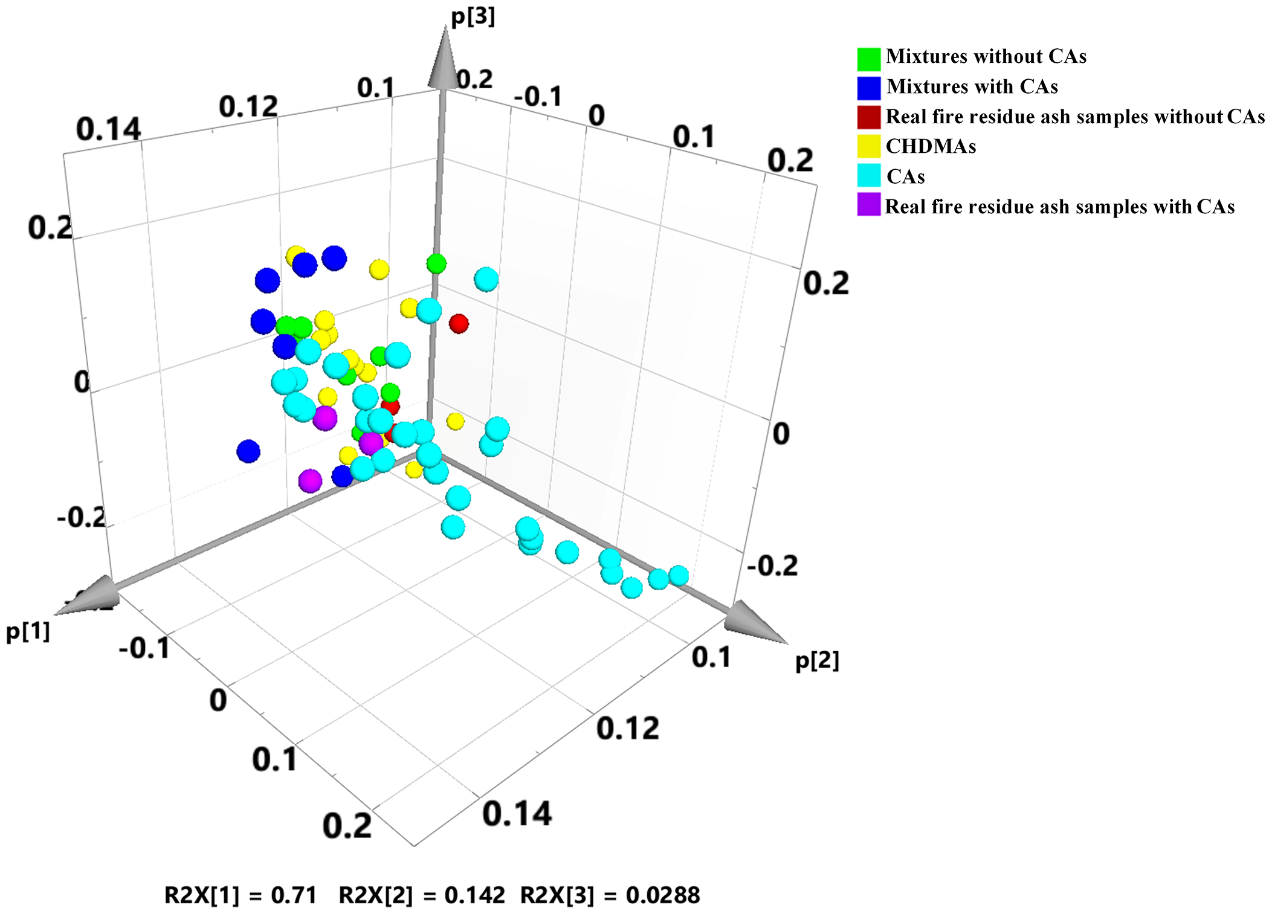


**Figure S5.** Three-dimensional scores plot with PC1, PC2 and PC3 of all samples studied.
